# Vaccination Programs for Endemic Infections: Modelling Real versus Apparent Impacts of Vaccine and Infection Characteristics

**DOI:** 10.1038/srep15468

**Published:** 2015-10-20

**Authors:** Romain Ragonnet, James M. Trauer, Justin T. Denholm, Nicholas L. Geard, Margaret Hellard, Emma S. McBryde

**Affiliations:** 1 Centre for Population Health, Burnet Institute, 85 Commercial Road, Melbourne 3004 Australia; 2 Victorian Infectious Diseases Service, Royal Melbourne Hospital, Grattan Street, Parkville 3050 Australia; 3 Department of Medicine (Royal Melbourne Hospital/Western Hospital), University of Melbourne, Victoria 3010 Australia; 4 Department of Microbiology and Immunology, University of Melbourne, Victoria 3010 Australia; 5 Victorian Tuberculosis Program, Melbourne Health, Melbourne, Victoria 3000 Australia; 6 Centre for Epidemiology and Biostatistics, MSPGH, University of Melbourne, Victoria 3053 Australia

## Abstract

Vaccine effect, as measured in clinical trials, may not accurately reflect population-level impact. Furthermore, little is known about how sensitive apparent or real vaccine impacts are to factors such as the risk of re-infection or the mechanism of protection. We present a dynamic compartmental model to simulate vaccination for endemic infections. Several measures of effectiveness are calculated to compare the real and apparent impact of vaccination, and assess the effect of a range of infection and vaccine characteristics on these measures. Although broadly correlated, measures of real and apparent vaccine effectiveness can differ widely. Vaccine impact is markedly underestimated when primary infection provides partial natural immunity, when coverage is high and when post-vaccination infectiousness is reduced. Despite equivalent efficacy, ‘all or nothing’ vaccines are more effective than ‘leaky’ vaccines, particularly in settings with high risk of re-infection and transmissibility. Latent periods result in greater real impacts when risk of re-infection is high, but this effect diminishes if partial natural immunity is assumed. Assessments of population-level vaccine effects against endemic infections from clinical trials may be significantly biased, and vaccine and infection characteristics should be considered when modelling outcomes of vaccination programs, as their impact may be dramatic.

Vaccination is a potentially powerful preventive response against endemic infections, with two major infectious diseases, smallpox in humans and rinderpest in bovines, having already been eradicated through vaccination campaigns^1^,^2^,^3^. However, the impact of vaccination programs has not always met expectations^4^,^5^,^6^, with its impact varying widely according to the setting in which it is introduced, making policy decision-making challenging^7^,^8^,^9^,^10^,^11^.

This variation in vaccine impact makes it difficult to predict the true population effect of a vaccination program. While the theoretical concept of vaccine efficacy describes the individual level benefit – how much less likely an individual is to acquire infection following a given exposure – clinical trials assess vaccine effectiveness at the population level. However, both may fail to capture the indirect effect of vaccination, due to reduced transmission to unvaccinated subjects in the wider population. This indirect effect is impossible to fully assess from clinical trial data alone. From a public health perspective, it is the overall effect (that is, a combination of both direct and indirect effects) that should be considered to fully evaluate an intervention’s impact. This composite effect of vaccination is not readily assessed by clinical trials, though some have tried to estimate it^12^,^13^, and so the direct effect is often presented as the measure of a vaccine’s efficacy^14^,^15^,^16^,^17^,^18^,^19^.

Some aspects of the complexity involved in assessment of the vaccine impact have been addressed previously, with prior work considering the impact of different study designs, as well as different indicators of vaccine effectiveness^20^,^21^. Longini *et al.*^22^ further highlighted the difficulty in finding the optimal design for assessing vaccine impact, due to the impact of factors such as vaccine efficacy and force of infection, and it has been shown that some configurations lead to incorrect estimates of vaccine effectiveness^23^.

Mathematical modelling provides an opportunity to consider the interaction between direct and indirect effects within a population, and to re-examine approaches to estimating vaccine effectiveness. Several modelling works considering different measures of effectiveness have highlighted the potential limitations of the concept of direct effectiveness^8^,^24^,^25^,^26^. Models also allow for consideration of various characteristics of the pathogen, vaccine and host immunity in order to identify modifiers of vaccine effect. Gomes *et al.* have previously modelled the impact of the level of natural immunity acquired from infection on the reduction of the disease burden^27^,^28^. Although both are important issues in assessing the impact of vaccines, heterogeneity of vaccine efficacy across recipients and the potential for inaccurate estimates are often studied separately, despite their interdependence having been described in relation to acute epidemics of measles and influenza^24^. Vaccination modelling consistently considers one of two types of vaccine: ‘leaky’^24^,^29^,^30^,^31^,^32^,^33^ or ‘all or nothing’ (‘AoN’)^24^,^25^,^33^,^34^,^35^,^36^,^37^. A ‘leaky’ vaccine provides the same partial reduction of susceptibility to every vaccinated individual, while an ‘AoN’ vaccine provides complete protection to a proportion of vaccinated individuals, with the remainder receiving no direct benefit. Despite the marked difference between these two approaches^38^,^39^, little previous work has explored the population level impact of these assumptions.

In this study, we present a general model of endemic infectious disease that is flexible to assumptions regarding vaccine leakiness, the presence and duration of latency and the degree of protection or increased susceptibility following a previous infection. We use this model to study the impact of an imperfect vaccine, which may produce either partial or complete immunity in vaccinated subjects, to assess both direct and indirect vaccine effectiveness. Next we consider the impact of re-infection on vaccine effectiveness, varying the degree of protection against repeat infection from complete immunity to increased susceptibility. We then highlight the discrepancies between the theoretical protection (vaccine efficacy), the observed impact (direct effectiveness) and the true outcome of vaccination programs (overall effectiveness). This approach allows consideration of a broad range of vaccination outcomes, in terms of both observed and population effects, using a model that is applicable to a broad range of vaccines and infections.

## Results

Table 1 shows the different parameters used in this model. Both baseline values and ranges are presented. The measures of effectiveness presented in this section correspond to a comparison between pre and post-vaccination equilibriums (see the Methods section for a full description of the effectiveness calculations).

### Individual parameter variation

**Figure 1:**
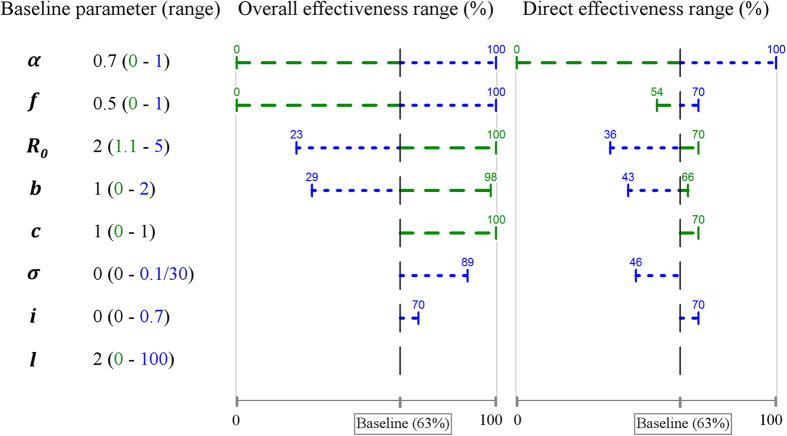
Results of individual parameter variations. The dotted blue range corresponds to an increase in the parameter value, whereas the dashed green range corresponds to a decrease. Vaccines are assumed to be ‘leaky’ at baseline while ‘AoN’ vaccines are considered when ***i*** = **0.7.**

The baseline configuration results in estimates of 63% for both overall and direct effectiveness with vaccine efficacy *α* assumed to be 70%. Figure 1 presents the impact of variation of a single parameter on overall and direct vaccine effectiveness with other parameters held at their fixed baseline values (see Table 1).

**Figure 2:**
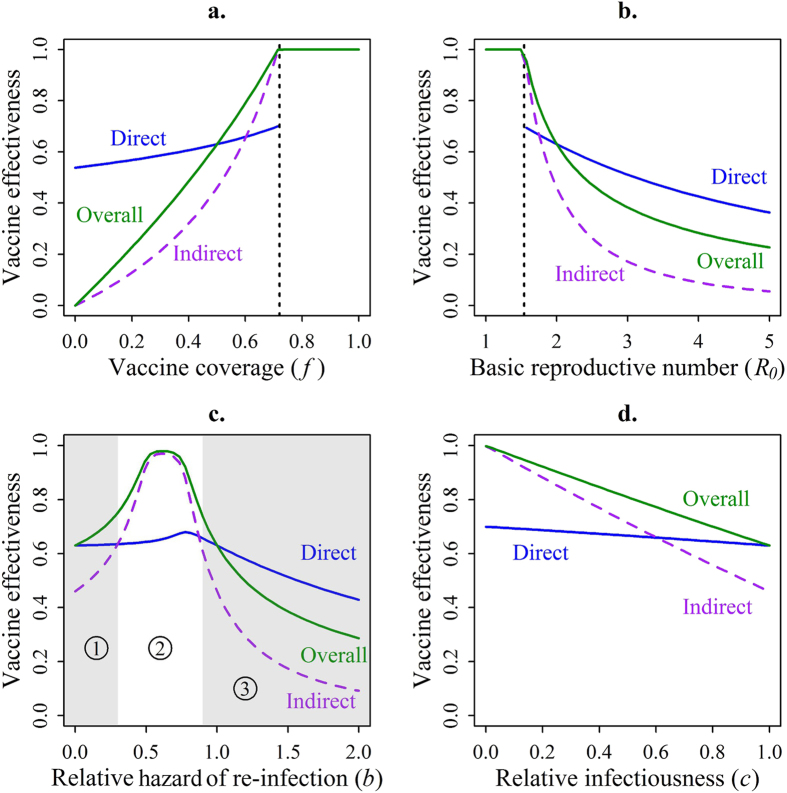
Impact of single parameter variations on the different measures of effectiveness. (**a**) Variation of the vaccine coverage. (**b**) Variation of the basic reproductive number. (**c**) Variation of the relative hazard of re-infection. (1) Low prevalence regardless presence of vaccination. (2) High prevalence with no vaccination whereas prevalence is still low in presence of vaccination. (3) High prevalence regardless presence of vaccination. (**d**) Variation of the relative infectiousness for vaccinated individuals. (**a**,**b**) The vertical dashed lines indicate a threshold in the parameter value after/before which a vaccination program would be totally effective (i.e. herd immunity). Direct effectiveness is not measurable in such settings, as the attack rate in vaccinated and unvaccinated individuals is zero.

As would be expected, the parameters with the greatest impact on vaccine effectiveness are vaccine efficacy *α* and vaccine coverage *f*. However, a marked difference between the impact on overall and direct effectiveness is observed as vaccine coverage is varied (Fig. 2a). As vaccine coverage increases, overall effectiveness increases markedly, while only a slight variation is seen in direct effectiveness. For example, a low vaccine coverage (*f* = 0.2) leads to a high value for direct effectiveness (57%), while the estimate of overall effectiveness is only 23%.

The basic reproductive number (*R*_0_) also strongly influences vaccine effectiveness, but, as for coverage, direct effectiveness is less affected than overall effectiveness (Fig. 2b). Furthermore, we note that the impact of a vaccination program would be underestimated for less transmissible infections (*R*_0_ < 2), since overall effectiveness is greater than direct effectiveness in such settings. By contrast, the impact would be overestimated for more infectious organisms (*R*_0_ > 2), with direct effectiveness greater than overall.

The risk of re-infection also has a strong influence on vaccine effectiveness (Fig. 2c). When considering a relative hazard of re-infection (*b*) either equal to 0 (total natural immunity) or equal to one (risk of infection unchanged after a primary episode), both direct and overall effectiveness are equal at 63%. However, at values representing partial natural immunity (0 < *b* < 1), overall effectiveness increases while direct effectiveness remains stable leading to an under-estimation of vaccine impact. For example, when assuming a partial natural immunity of 40% (*b* = 0.6), overall effectiveness is 98% while direct effectiveness is only 65%. By contrast, if we consider a higher risk of re-infection (*b* > 1) after primary infection, as may be seen with sexually transmitted diseases, the population effect will be over-estimated.

The peak in overall effectiveness occurring at values for *b* of around 0.7 (Fig. 2c, area ②) arises from disease prevalence reaching moderate levels in the unvaccinated population, while prevalence remains much lower in the vaccinated population. That is, in the vaccinated population, disease prevalence reaches very low levels around the transition between areas ① and ② while in the unvaccinated population this occurs later – around the transition between areas ② and ③. By contrast, outside of this range, prevalence in the respective populations is more comparable.

Direct effectiveness is relatively insensitive to variations of relative infectiousness for vaccinated individuals *c*, whereas overall effectiveness is more responsive to this parameter (Fig. 2d). That is, reduction of infectiousness (*c* < 1) can lead to a substantial increase in overall effectiveness with only a slight rise in direct effectiveness, resulting in underestimation of vaccine impact.

### Vaccine leakiness

With the baseline parameters, we note that an ‘AoN’ vaccine is slightly more effective than a ‘leaky’ one when measuring overall effectiveness (Fig. 1, 70% vs. 63%). However, when other parameters are varied, the gap between the two types of vaccine varies widely.

**Figure 3:**
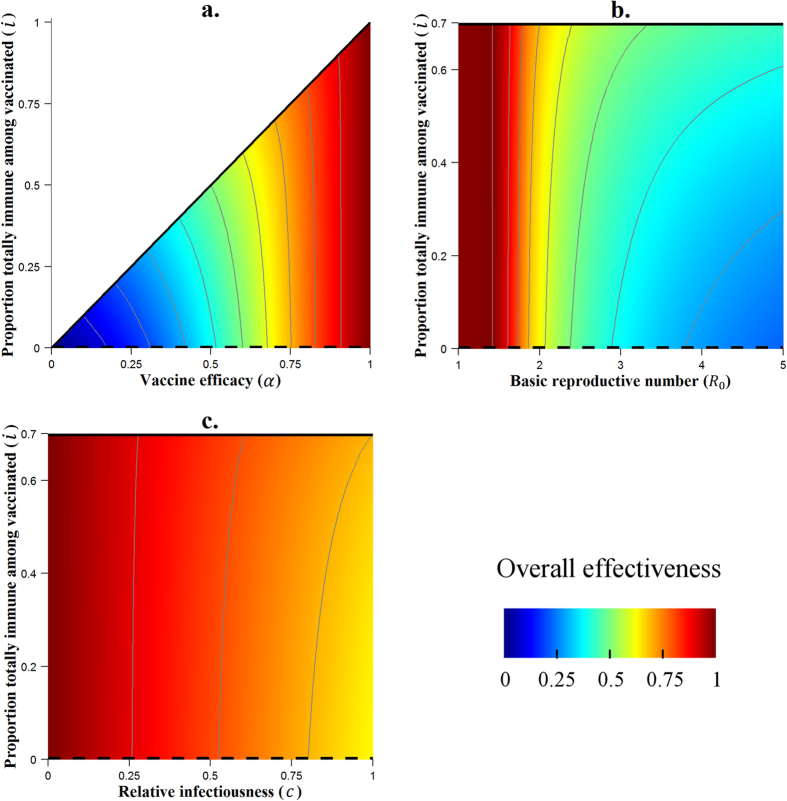
Results of the variation of the vaccine mechanism (through the parameter *i*) paired with variations of other parameters (*α*, *R*_0_ and *c*) on overall effectiveness. The thin grey contour lines connect points at which overall effectiveness takes the same value, with each successive line separated by a difference of 10% in effectiveness. Continuous black lines indicate the ‘all or nothing’ vaccine configuration, while dashed black lines correspond to fully ‘leaky’ vaccines. In panel (**a**) the value of ***i*** is bounded by the vaccine efficacy ***α***, with the diagonal of the graph corresponding to the ‘all or nothing’ configuration.

For a low vaccine efficacy (*α* = 0.2), an ‘AoN’ vaccine has a much greater overall effectiveness than a ‘leaky’ vaccine (20% vs. 12%), while in high efficacy settings (*α* = 0.9) both types of vaccine lead to equivalent overall effectiveness. (Fig. 3a)

When the infection is less transmissible (*R*_0_ = 1.75), overall effectiveness of both ‘AoN’ and ‘leaky’ vaccines are similar, whereas with a more infectious organism (*R*_0_ = 5) an ‘AoN’ vaccine leads to a better overall effectiveness than a ‘leaky’ one (44% vs. 23%). (Fig. 3b)

Under the assumption that the vaccine does not reduce infectiousness for breakthrough infections (*c* = 1, baseline configuration), an ‘AoN’ vaccine has a greater impact than a ‘leaky’ one (70% vs. 63%, overall effectiveness). However, if a strong reduction in the infectiousness for these individuals is incorporated (*c* = 0.25), both types of vaccine are equally effective at the overall level. (Fig. 3c)

### Risk of re-infection

As variation in the relative hazard of re-infection (*b*) led to large changes in overall effectiveness when varied individually, we considered the impact of varying this parameter in combination with a second parameter.

**Figure 4:**
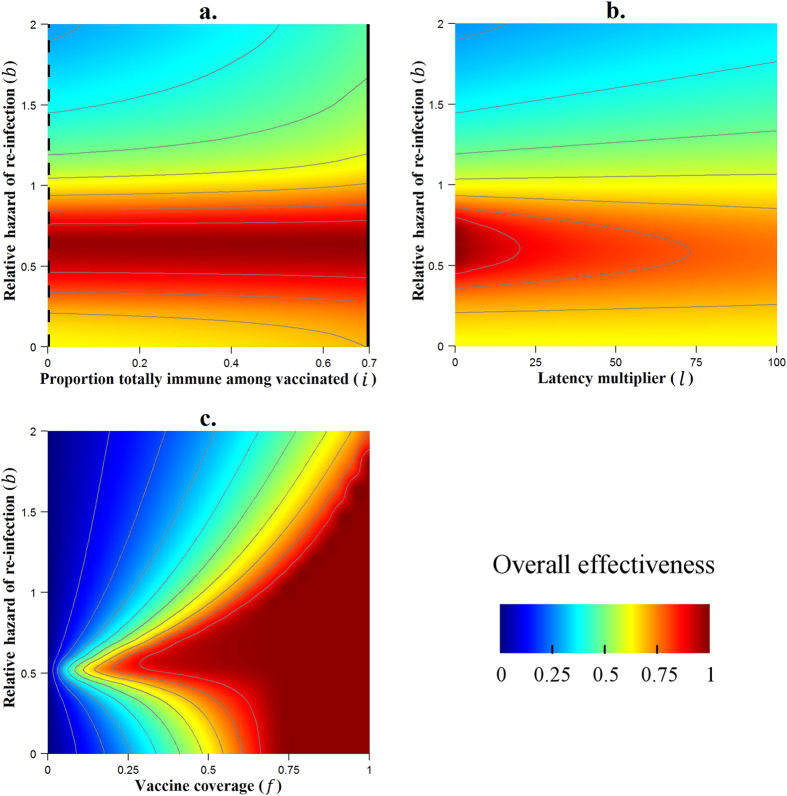
Results of the variation of the risk of re-infection (*b*) paired with variations of other parameters (*i*, *l* and *f*) on overall effectiveness. The thin grey contour lines connect points at which overall effectiveness takes the same value, with each successive line separated by a difference of 10% in effectiveness. Continuous black lines indicate the ‘all or nothing’ vaccine configuration, while dashed black lines correspond to fully ‘leaky’ vaccines. In panels (**b**,**c**) a ‘leaky’ vaccine is considered.

Figure 4a shows that the difference between a ‘leaky’ and an ‘AoN’ vaccine is conditioned by the relative hazard of re-infection. Indeed, if the relative hazard of re-infection is around 0.6, a ‘leaky’ vaccine and an ‘AoN’ vaccine are equally effective, with an overall effectiveness reaching 98% in both situations. However, if the risk of infection is twice as high after primary infection (*b* = 2), the ‘AoN’ vaccine is much more effective than the ‘leaky’ one (47% vs. 29%, overall effectiveness). For infections which involve a high natural immunity (*b* close to 0), the ‘AoN’ vaccine leads once again to a higher overall effectiveness, although the difference is slight compared to the ‘leaky’ vaccine.

When varied singly, the latency parameter *l* had no impact on overall effectiveness. Figure 4b shows that variation of this parameter can have an impact when the risk of re-infection is varied (*b* ≠ 1). For a situation where the risk of re-infection is low (*b* < 1), the introduction of a latency period leads to an attenuation of the overall impact of the vaccine. By contrast, when the risk of re-infection is high (*b* > 1), a latency period increases overall effectiveness. The same results are observed when considering an ‘AoN’ vaccine instead of a ‘leaky’ vaccine.

The vaccine coverage *f* is one of the parameters with the greatest impact on the vaccine effectiveness. We noted on the Fig. 2a that there is a threshold for *f* after which the vaccine is totally effective (*f* = 0.72 with other parameters at baseline). Figure 4c shows how much this threshold could be reduced or increased when we consider different risks of re-infection. Thus, when the relative hazard of re-infection is 0.6, vaccine coverage of 0.65 is sufficient to achieve eradication, but with a high relative hazard of 2, even complete coverage (*f* = 1) is insufficient to achieve this. This result remains valid for both ‘AoN’ and ‘leaky’ vaccines.

## Discussion

Our study highlights the challenges of assessing vaccine impact and reinforces that the potential effectiveness of vaccination programs is poorly characterized by efficacy alone. We present a general model of vaccination for infectious disease, so that our conclusions are applicable to a broad range of infections and pre-exposure vaccines. The structure and parameterization of our model allows us to demonstrate several sources of heterogeneity and bias for vaccine impact estimates, and highlights that the measurable effect, even from well-designed clinical trials, might not be a reliable measure of population level impact.

Our model incorporates different mechanisms of vaccine efficacy, including a continuous transition between the two extremes of ‘leaky’ and ‘all or nothing’ (‘AoN’) vaccines. The model flexibility permits the simultaneous consideration of multiple modifiers of vaccine effectiveness under different assumptions regarding the vaccine mechanism.

We demonstrate that ‘AoN’ vaccines are consistently at least as effective as ‘leaky’ vaccines, but prove to be much more effective under conditions where diseases are more difficult to eradicate. In particular, ‘AoN’ vaccines are most beneficial when risk of re-infection is high, when the infection is highly transmissible or when the vaccine has low efficacy. Even though it is difficult to determine which mechanism is associated with a given vaccine in practice, this study provides insights into why population-level vaccination failure may occur despite significant biological efficacy. ‘Leaky’ vaccines in particular may have an overall effectiveness that is significantly lower than the vaccine efficacy in certain configurations. Furthermore we demonstrate that the impact of such assumptions regarding vaccine mechanism are not trivial, suggesting that it may be worthwhile considering different scenarios when modelling vaccination against infectious diseases.

We find risk of re-infection to be an important modifier of vaccine effectiveness. This observation agrees with and extends other modelling studies focusing on the partial immunity acquired after infection associated with a ‘leaky’ vaccine^27^,^28^, however this finding has not previously been tested under varying assumptions regarding the mechanism of vaccine protection. We particularly notice that a vaccine is most effective when individuals acquire partial natural immunity after a primary infection and that, surprisingly, the relative impact of vaccination is increased compared to a situation in which infection results in total natural immunity. Varying the risk of re-infection also reveals the significant impact of latency duration on vaccine impact, which is not appreciated on univariate analysis. We find that long latency periods increase vaccine effectiveness when the risk of re-infection is high, whereas effectiveness is attenuated in the setting of partial natural immunity. These conclusions demonstrate the need to properly assess re-infection dynamics in order to forecast vaccine impact accurately. Our observations concerning the impact of long latency could be particularly relevant to diseases such as tuberculosis, although more focused study is needed. Indeed, our current model structure would need to be adapted in order to be relevant to tuberculosis, as more complex latency structures are typically employed, as well as the possibility of re-infection during latency^40^.

For some diseases, vaccine trials are generally undertaken in highly endemic settings in order to minimize sample size required. However, we have shown that vaccine effectiveness can be dramatically affected when diseases have a high basic reproductive number. This observation suggests that estimates obtained in such trials could systematically underestimate the effect of a vaccine in lower burden settings.

The different results for direct and overall vaccine effectiveness highlight the potential gap between the measured effect and the true population effect. Underestimation of vaccine impact is particularly pronounced when a primary infection confers partial natural immunity. This source of bias would be particularly important for a disease like pertussis which produces partial natural immunity after infection, particularly given that this degree of immunity wanes with time^41^. This finding about partial immunity recalls the relation established by Fine *et al.* between the apparent vaccine effectiveness and the degree of heterologous exposure of the population^42^. Indeed, when the latter effect was assumed to provide partial protection to exposed individuals, observed vaccine effectiveness was reduced. When studied under conditions of high vaccine coverage, vaccine effect could be dramatically underestimated, because the unvaccinated group become protected by herd immunity. This result is consistent with previous modelling studies^8^,^24^, and demonstrates that assessments from large vaccination campaigns might be overly pessimistic.

One limitation of this study is that we have focused on comparison of pre and post-vaccination equilibriums. This choice is justified by the fact that the infection dynamics after vaccine introduction are highly dependent on the mode of implementation of the intervention which is dependent on the vaccine itself. Future applied works based on this theoretical study may include a finite time-frame after vaccine introduction for effectiveness calculation in order to generate estimates about vaccination programs of specific duration.

Given that we considered equilibriums for calculating the vaccine effectiveness, we tried to find analytic expressions for the non-trivial solutions of the steady state of the system. Unfortunately, such solutions of the non-linear system could not be obtained even with the assistance of computer algebra software, due to the large number of variables and parameters that directly impact these outcomes in our model and therefore, such solutions would likely be unwieldy if they were obtained. While we acknowledge this limitation to our findings, the ability to simultaneously consider the impact of several key parameters for infectious disease transmission is also the major strength of the numerical results presented.

The findings emphasised by this theoretical study will be reinforced with more applied modelling studies in future work. In particular, we will parameterise the model to individual diseases and adapt the model structure where necessary to observe how our conclusions must be modified for specific infections. Further, our modelling could be applied in parallel with clinical vaccine trials to better predict the true impact of a vaccine. A recent study from Lehtinen *et al.* on human papillomavirus vaccination illustrates the possibility of estimating herd immunity from clinical trials data^43^. In summary, this study provides insights into the true impact of vaccination programs under various conditions and highlights relatively common situations in which this impact could be underestimated. It also demonstrates that clinical trials measuring direct effectiveness may be poor estimates of the true effectiveness of a vaccine program. Mathematical modelling can help to improve accuracy in estimates of vaccine effectiveness, as well to optimize the design of both vaccine clinical trials and public health interventions.

## Methods

### Model structure

**Figure 5:**
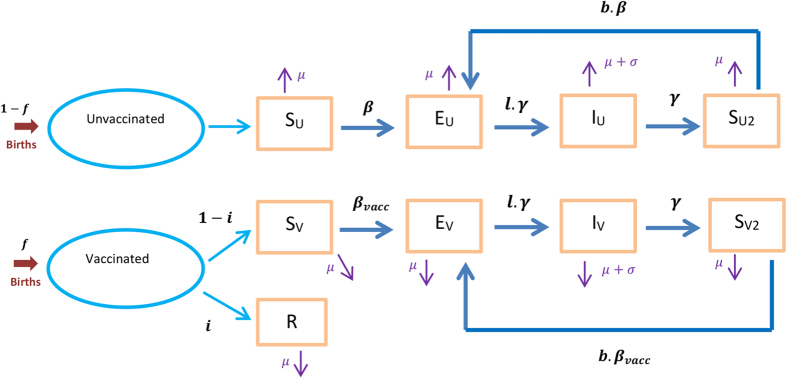
Structure of the compartmental model. The rectangular boxes represent the different categories in which the population is structured. The dark blue arrows stand for the transitions that occur between the different categories. The birth and death flows are represented by the red and purple arrows respectively.

We use a frequency-dependent dynamic compartmental model of infectious disease to estimate the impact of a vaccination program, with individuals classified by both disease and vaccination state (Fig. 5). In all simulations, fully protected individuals are denoted (‘*R*’), while individuals who did not receive complete vaccine protection are grouped by vaccination status (‘*U*’ and ‘*V’* subscripts), and into four disease compartments, namely susceptible (‘*S*’), exposed (‘*E*’), infectious (‘*I*’) and post-infection (‘*S*_*2*_’). The total population is denoted *N*, such that


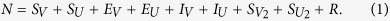


### Demographic processes

Births and deaths are included in the model, with deaths not due to disease occurring at the same rate (‘*μ*’) in every compartment, while an additional disease-specific mortality rate (‘*σ*’) is applied to the *I*_*V*_ and *I*_*U*_ compartments. In order to maintain a closed population and a constant population size *N*, a new birth is introduced into the susceptible compartment for every death.

### Transmission dynamics

We assume that fully susceptible individuals are exposed to infection through the transmission rate (‘*β*’). A variable proportion (‘*i*’) of vaccinated individuals derive complete protection, while the remaining proportion (1 − *i*) are conferred a reduction in the risk of infection 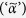. 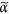 is calculated to maintain the target vaccine efficacy of *α*, through the equation 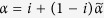. At one extreme, with the parameter *i* equal to *α* and 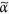 equal to zero an ‘AoN’ vaccine is modelled. In contrast with *i* = 0, the vaccine is entirely ‘leaky’, with every vaccinated individual having a reduction in the risk of infection 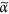 equal to *α*. This latter configuration is considered at baseline. In either case, the transmission rate for vaccinated individuals who are incompletely protected is 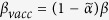. Duration of infectiousness (‘*γ*’), latency multiplier (‘*l*’) and natural immunity (‘*b*’) are unaffected by vaccination status, with the natural immunity parameter permitting a modified risk of re-infection by comparison to primary infection. Similarly, the multiplier *c* allows modification of infectiousness for vaccinated infected individuals (see eqs. (3) and (8)).

### Differential equations

The differential equations for unvaccinated individuals are given by:


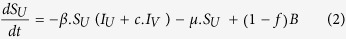



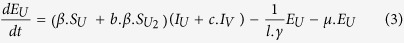



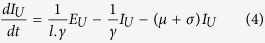



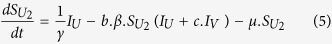


where


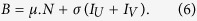


The differential equations for vaccinated individuals are given by:


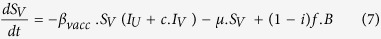



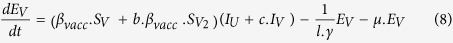



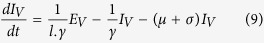



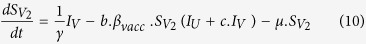



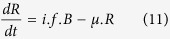


We used the software R version 3.1.2 with the package deSolve version 1.11 to obtain numerical solutions to this system of differential equations.

### Effectiveness calculation

**Figure 6:**
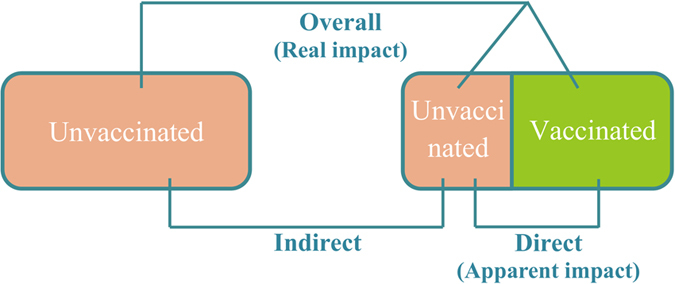
Different measures of vaccine effectiveness. Effectiveness is estimated by comparing disease incidences across the different population groups. The left and right boxes represent simulation scenarios with and without vaccination. All are measured in the model. Direct effectiveness is assessed in clinical trials. Overall effectiveness is the most important from a public health perspective.

We measure three estimates of vaccine effectiveness by comparing pre- (*f* = 0) and post-vaccination equilibriums (Fig. 6). We use attack rates to calculate the different indicators of vaccine effectiveness, obtained by dividing the number of new cases per year by the size of the corresponding population. Three attack rates are calculated: *AR*_0_ corresponds to the attack rate in the pre-vaccination population at equilibrium, while *AR*_*V*_ and *AR*_*U*_ correspond to the attack rate for vaccinated and unvaccinated populations at equilibrium after vaccine introduction. Therefore, we define the direct, indirect and overall vaccine effectiveness as follows:


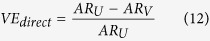



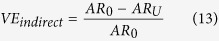



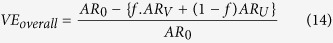


We focus on direct (*VE*_*direct*_) and overall effectiveness (*VE*_*overall*_), as these represent the apparent impact and the overall impact of vaccination respectively.

#### Calculation of the transmission rate *β*

As the transmission rate can be difficult to estimate for many infectious diseases, we chose to not attribute any single value to *β* to the model. Instead, *β* is calculated from the other parameters, through the equation for the basic reproductive number:


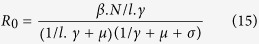


By rearranging equation (15), we obtain the following expression for *β*:


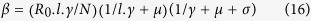


#### Parameter variation

Only two parameters are fixed: the natural death rate *μ* (16/1000 person/year, i.e. 62.5 years life expectancy) and the infectiousness duration *γ* (1 month). The other parameters are varied singly and in pairs to identify interactions with the potential to affect vaccine effectiveness. For each simulation, every parameter not being analyzed is held at a fixed baseline value. The baseline value and range of variation of each parameter are presented in Table 1. Note that the proportion of individuals totally immunized after vaccination cannot exceed vaccine efficacy (i.e. *i* is bounded by *α*).

For the latency multiplier *l*, the absence of latency is approximated by a very low value (*l* = 10^−10^) since a null value is not admissible in the model (see eqs. (2), (3), (6) and (7)).

## Additional Information

**How to cite this article**: Ragonnet, R. *et al.* Vaccination Programs for Endemic Infections: Modelling Real versus Apparent Impacts of Vaccine and Infection Characteristics. *Sci. Rep.* **5**, 15468; doi: 10.1038/srep15468 (2015).

## Figures and Tables

**Table 1:** Baseline parameter values and range of variation analysed.

Parameter	Description	Baseline Value	Considered range
Disease related			
1/γ	Mean duration of infectiousness	1 month	Fixed
*l*	Relative duration of latency compared to the infectiousness duration	2	10^−10^ − 100
*R*_0_	Basic reproductive number	2	1 − 5
*b*	Relative hazard of secondary infection	1	0 − 2
*σ*	Infection-relative death rate	0	0 − 0.1 (/person/month)
Vaccine related			
*α*	Vaccine efficacy	0.7	0 − 1
*f*	Vaccine coverage	0.5^a^	0 − 1^a^
*i*	Proportion of vaccinated individuals totally immunized	0	0 − *α*^b^
*c*	Relative infectiousness of vaccinated infectious individuals	1	0 − 1
Demographic			
*μ*	Natural annual death rate	16/1 000 persons/year i.e. 62.5 years life expectancy	Fixed

^a^Vaccine coverage used for the post-vaccination phase, as this is null in the pre-vaccine phase.

^b^The proportion of individuals completely protected after vaccination cannot exceed vaccine efficacy.

## References

[b1] Greenwood B. The contribution of vaccination to global health: past, present and future. Philos Trans R Soc Lond B Biol Sci 369, 20130433 (2014). 24821919 10.1098/rstb.2013.0433 PMC4024226

[b2] Moutou F. The second eradication: Rinderpest. Bull Soc Pathol Exot 107, 137–138 (2014). 24566884 10.1007/s13149-014-0336-y

[b3] Fenner F. A successful eradication campaign. Global eradication of smallpox. Rev Infect Dis 4, 916–930 (1982). 6293036 10.1093/clinids/4.5.916

[b4] Wilson A. T., Henderson I. R., Moore E. J. & Heywood S. N. Whooping-Cough: Difficulties in Diagnosis and Ineffectiveness of Immunization. Br Med J 2, 623–626 (1965). 14331622 10.1136/bmj.2.5462.623 PMC1846011

[b5] Najera J. A., Gonzalez-Silva M. & Alonso P. L. Some lessons for the future from the Global Malaria Eradication Programme (1955-1969). PLoS Med 8, e1000412 (2011). 21311585 10.1371/journal.pmed.1000412 PMC3026700

[b6] Poland G. A. Pertussis outbreaks and pertussis vaccines: new insights, new concerns, new recommendations? Vaccine 30, 6957–6959 (2012). 23141958 10.1016/j.vaccine.2012.09.084

[b7] Mangtani P. *et al.* Protection by BCG vaccine against tuberculosis: a systematic review of randomized controlled trials. Clinical infectious diseases: an official publication of the Infectious Diseases Society of America 58, 470–480 (2014). 24336911 10.1093/cid/cit790

[b8] Haber M. Estimation of the direct and indirect effects of vaccination. Statistics in medicine 18, 2101–2109 (1999). 10441766 10.1002/(sici)1097-0258(19990830)18:16<2101::aid-sim178>3.0.co;2-6

[b9] Wang H. *et al.* Meta-analysis of vaccine effectiveness of mumps-containing vaccine under different immunization srategies in China. Vaccine 32, 4806–4812 (2014). 25000591 10.1016/j.vaccine.2014.05.061

[b10] Colditz G. A. *et al.* Efficacy of BCG vaccine in the prevention of tuberculosis. Meta-analysis of the published literature. Jama 271, 698–702 (1994). 8309034

[b11] Tazhibi M., Hajivandi A., Tafti A. D. & Fallahzadeh H. The efficacy of hepatitis B vaccine in Iranian population: A systematic review and meta-analysis. J Educ Health Promot 3, 53 (2014). 25077146 10.4103/2277-9531.134741 PMC4114169

[b12] Pannaraj P. S. *et al.* School-Located Influenza Vaccination Decreases Laboratory-Confirmed Influenza and Improves School Attendance. Clin Infect Dis 59, 325–332 (2014). 24829215 10.1093/cid/ciu340 PMC4155443

[b13] Vestrheim D. F. *et al.* Indirect effect of conjugate pneumococcal vaccination in a 2+1 dose schedule. Vaccine 28, 2214–2221 (2010). 20056192 10.1016/j.vaccine.2009.12.054

[b14] Harper D. M. *et al.* Sustained efficacy up to 4.5 years of a bivalent L1 virus-like particle vaccine against human papillomavirus types 16 and 18: follow-up from a randomised control trial. Lancet 367, 1247–1255 (2006). 16631880 10.1016/S0140-6736(06)68439-0

[b15] Skinner S. R. *et al.* Efficacy, safety, and immunogenicity of the human papillomavirus 16/18 AS04-adjuvanted vaccine in women older than 25 years: 4-year interim follow-up of the phase 3, double-blind, randomised controlled VIVIANE study. Lancet 384, 2213–2227 (2014). 25189358 10.1016/S0140-6736(14)60920-X

[b16] Hildesheim A. *et al.* Efficacy of the HPV-16/18 vaccine: final according to protocol results from the blinded phase of the randomized Costa Rica HPV-16/18 vaccine trial. Vaccine 32, 5087–5097 (2014). 25018097 10.1016/j.vaccine.2014.06.038 PMC4166498

[b17] Zhu F. C. *et al.* Efficacy, immunogenicity and safety of the HPV-16/18 AS04-adjuvanted vaccine in healthy Chinese women aged 18-25 years: results from a randomized controlled trial. International journal of cancer. Journal international du cancer 135, 2612–2622 (2014). 24740596 10.1002/ijc.28897 PMC4277330

[b18] Bonten M. J. *et al.* Polysaccharide conjugate vaccine against pneumococcal pneumonia in adults. N Engl J Med 372, 1114–1125 (2015). 25785969 10.1056/NEJMoa1408544

[b19] Tregnaghi M. W. *et al.* Efficacy of pneumococcal nontypable Haemophilus influenzae protein D conjugate vaccine (PHiD-CV) in young Latin American children: A double-blind randomized controlled trial. PLoS Med 11, e1001657 (2014). 24892763 10.1371/journal.pmed.1001657 PMC4043495

[b20] Halloran M. E., Struchiner C. J. & Longini I. M. Jr. Study designs for evaluating different efficacy and effectiveness aspects of vaccines. Am J Epidemiol 146, 789–803 (1997). 9384199 10.1093/oxfordjournals.aje.a009196

[b21] Halloran M. E., Longini I. M. Jr. & Struchiner C. J. Design and interpretation of vaccine field studies. Epidemiologic reviews 21, 73–88 (1999). 10520474 10.1093/oxfordjournals.epirev.a017990

[b22] Longini I. M. Jr., Sagatelian K., Rida W. N. & Halloran M. E. Optimal vaccine trial design when estimating vaccine efficacy for susceptibility and infectiousness from multiple populations. Stat Med 17, 1121–1136 (1998). 9618773 10.1002/(sici)1097-0258(19980530)17:10<1121::aid-sim824>3.0.co;2-e

[b23] Halloran M. E., Longini I. M. Jr. & Struchiner C. J. Estimability and interpretation of vaccine efficacy using frailty mixing models. Am J Epidemiol 144, 83–97 (1996). 8659489 10.1093/oxfordjournals.aje.a008858

[b24] Shim E. & Galvani A. P. Distinguishing vaccine efficacy and effectiveness. Vaccine 30, 6700–6705 (2012). 22944629 10.1016/j.vaccine.2012.08.045 PMC3798059

[b25] Baussano I., Garnett G., Segnan N., Ronco G. & Vineis P. Modelling patterns of clearance of HPV-16 infection and vaccination efficacy. Vaccine 29, 1270–1277 (2011). 21145375 10.1016/j.vaccine.2010.11.082

[b26] Becker N. G., Britton T. & O’Neill P. D. Estimating vaccine effects on transmission of infection from household outbreak data. Biometrics 59, 467–475 (2003). 14601747 10.1111/1541-0420.00056

[b27] Gomes M. G., White L. J. & Medley G. F. Infection, reinfection, and vaccination under suboptimal immune protection: epidemiological perspectives. J Theor Biol 228, 539–549 (2004). 15178201 10.1016/j.jtbi.2004.02.015

[b28] Gomes M. G., Franco A. O., Gomes M. C. & Medley G. F. The reinfection threshold promotes variability in tuberculosis epidemiology and vaccine efficacy. Proc Biol Sci 271, 617–623 (2004). 15156920 10.1098/rspb.2003.2606 PMC1691632

[b29] Hogea C., van Effelterre T. & Acosta C. J. A basic dynamic transmission model of Staphylococcus aureus in the US population. Epidemiology and infection 142, 468–478 (2014). 23701989 10.1017/S0950268813001106 PMC3915753

[b30] Ribassin-Majed L., Lounes R. & Clemencon S. Deterministic modelling for transmission of Human Papillomavirus 6/11: impact of vaccination. Math Med Biol 31, 125–49 (2013). 23475425 10.1093/imammb/dqt001 PMC4609570

[b31] Ejima K., Aihara K. & Nishiura H. The impact of model building on the transmission dynamics under vaccination: observable (symptom-based) versus unobservable (contagiousness-dependent) approaches. PloS one 8, e62062 (2013). 23593507 10.1371/journal.pone.0062062 PMC3625221

[b32] Shim E. & Galvani A. P. Impact of transmission dynamics on the cost-effectiveness of rotavirus vaccination. Vaccine 27, 4025–4030 (2009). 19389452 10.1016/j.vaccine.2009.04.030

[b33] Kribs-Zaleta C. M. & Velasco-Hernandez J. X. A simple vaccination model with multiple endemic states. Math Biosci 164, 183–201 (2000). 10748286 10.1016/s0025-5564(00)00003-1

[b34] Lanzieri T. M., Bialek S. R., Ortega-Sanchez I. R. & Gambhir M. Modeling the potential impact of vaccination on the epidemiology of congenital cytomegalovirus infection. Vaccine 32, 3780–6 (2014). 24837782 10.1016/j.vaccine.2014.05.014 PMC4843116

[b35] Vanska S. *et al.* Impact of vaccination on 14 high-risk HPV type infections: a mathematical modelling approach. PloS one 8, e72088 (2013). 24009669 10.1371/journal.pone.0072088 PMC3756967

[b36] Lugner A. K., van Boven M., de Vries R., Postma M. J. & Wallinga J. Cost effectiveness of vaccination against pandemic influenza in European countries: mathematical modelling analysis. BMJ 345, e4445 (2012). 22791791 10.1136/bmj.e4445 PMC3395306

[b37] Choi Y. H. *et al.* Transmission dynamic modelling of the impact of human papillomavirus vaccination in the United Kingdom. Vaccine 28, 4091–4102 (2010). 19909831 10.1016/j.vaccine.2009.09.125

[b38] Halloran M. E., Haber M. & Longini I. M. Jr. Interpretation and estimation of vaccine efficacy under heterogeneity. Am J Epidemiol 136, 328–343 (1992). 1415152 10.1093/oxfordjournals.aje.a116498

[b39] Rahman M. S. Estimating vaccine efficacy under the heterogeneity of vaccine action in a nonrandomly mixing population. J Biopharm Stat 23, 394–412 (2013). 23437946 10.1080/10543406.2011.616974 PMC4130230

[b40] Trauer J. M., Denholm J. T. & McBryde E. S. Construction of a mathematical model for tuberculosis transmission in highly endemic regions of the Asia-Pacific. Journal of theoretical biology 358, 74–84 (2014). 24878110 10.1016/j.jtbi.2014.05.023

[b41] Campbell P. *et al.* Increased population prevalence of low pertussis toxin antibody levels in young children preceding a record pertussis epidemic in Australia. PLoS One 7, e35874 (2012). 22558249 10.1371/journal.pone.0035874 PMC3338806

[b42] Fine P. E. & Vynnycky E. The effect of heterologous immunity upon the apparent efficacy of (e.g. BCG) vaccines. Vaccine 16, 1923–1928 (1998). 9796044 10.1016/s0264-410x(98)00124-8

[b43] Lehtinen M. *et al.* Characteristics of a cluster-randomized phase IV human papillomavirus vaccination effectiveness trial. Vaccine 33, 1284–1290 (2015). 25593103 10.1016/j.vaccine.2014.12.019

